# A Case of Iatrogenic Cushing's Syndrome following Use of an Over-the-Counter Arthritis Supplement

**DOI:** 10.1155/2023/4769258

**Published:** 2023-03-11

**Authors:** Colin Dunn, Joshua Amaya, Patrick Green

**Affiliations:** University of Texas Southwestern Medical Center, Department of Internal Medicine, 5323 Harry Hines Blvd, Dallas, TX 75390, USA

## Abstract

**Background:**

Iatrogenic Cushing's syndrome is commonly seen as a complication of chronic steroid use. While most often associated with the use of prescription oral steroids, rare cases result from unintentional steroid exposure. In particular, numerous complementary and alternative medicines have been found to contain steroids not previously known to users. *Case Presentation*. Here, we present a case of iatrogenic Cushing's syndrome caused by prolonged ingestion of dexamethasone found within an over-the-counter arthritis supplement called Artri King.

**Conclusion:**

A thorough history of medication use to include over-the-counter medications and supplements may be required to identify the source of exogenous glucocorticoids in iatrogenic Cushing's syndrome.

## 1. Introduction

Cushing's syndrome describes a collection of clinical features that results from chronic exposure to supraphysiologic levels of glucocorticoids. It is most recognizable by the characteristic physical features of moon facies, an enlarged dorsocervical fat pad, easy bruisability, and truncal obesity; however, patients with Cushing's syndrome are otherwise at risk for developing a host of systemic disturbances including hypertension, glucose intolerance, decreased bone density, and menstrual irregularities [1].

While Cushing's syndrome can rarely result from endogenous hypercortisolism due to inappropriate production of either CRH, ACTH, or cortisol, usually due to hypersecretory lesions in the anterior pituitary or adrenal cortex, it is most commonly caused by the therapeutic administration of exogenous glucocorticoids. Cushing's syndrome caused by chronic glucocorticoid use, also known as iatrogenic Cushing's syndrome, is most often associated with the use of prescription oral steroids as might be used for the treatment of autoimmune or inflammatory disease. Rare instances are described of patients developing iatrogenic Cushing's syndrome due to accidental ingestion of glucocorticoids [2, 3].

Here, we report a case of iatrogenic Cushing's syndrome caused by glucocorticoids found in an over-the-counter arthritis supplement produced in Mexico called Artri King which is known to the FDA to contain undeclared amounts of dexamethasone.

## 2. Case Presentation

A 35-year-old Hispanic female presented to the emergency department with a chief complaint of progressive exertional dyspnea and upper abdominal pain. Her past medical history was notable for rheumatoid arthritis and a prior pulmonary embolism. She did not take any prescription medications. She was admitted to the hospital for severe anemia requiring blood transfusion due to a bleeding gastric ulcer which was ultimately attributed to heavy NSAID use and *H. pylori* infection. She was ultimately discharged home on *H. pylori* therapy, an iron supplement, and a plan to repeat upper endoscopy as an outpatient.

Tangential to this reason for admission, however, she was noted on arrival to display numerous Cushingoid features including moon facies, an enlarged dorsocervical fat pad, truncal obesity, easy bruisability, non-healing wounds, and oligomenorrhea. On presentation she was afebrile with HR 81, BP 134/85, SpO_2_ 100% on room air, with weight 66.2 kg; little comparison data was available, but her weight three years prior was 59.2 kg. Her initial lab work was notable for a hemoglobin 6.7 g/dL (reference values in parentheses; 11.2–15.7) with MCV 72 fL (79.4–94.8), a mild, chronic neutrophilia with associated left shift, and unremarkable basic chemistries including a sodium of 141 mmol/L (135–145) and potassium of 3.6 mmol/L (3.6–5.0); hemoglobin A1C was 5.6%. Imaging obtained during her stay incidentally revealed several old, atraumatic fractures of her ribs and feet with evidence of osteopenia.

A careful review of her home medications revealed that she had been taking a supplement from Mexico called Artri King for the past three years to alleviate her joint pains. This supplement was purchased at a store specializing in natural medicines from Mexico. She took an average of 6 of these pills a day. An internet search of this supplement yielded an FDA report indicating that it contained an undisclosed and unspecified amount of dexamethasone.

Despite the concern for hypercortisolism, testing for endogenous glucocorticoid excess was not pursued given the compelling history of exogenous glucocorticoid exposure. Instead, an ACTH stimulation test was conducted to assess the adequacy of her endogenous glucocorticoid production. This showed a low ACTH at <1.5 pg/mL (7.2–63.3) with a low 8 AM cortisol at <1.0 mcg/dL (5–23) and measurements of 3.2 and 3.7 mcg/dL at 30 minutes and 60 minutes, respectively, after administration of 250 mcg of cosyntropin; these findings were consistent with secondary adrenal insufficiency. Synthetic glucocorticoid screening was not pursued. A biopsy was performed of a nonhealing ulcer on her calf which revealed nonspecific ulceration; the consulting dermatology service thought that this ulcer was most likely a consequence of poor wound healing from her chronic steroid use.

In light of the abovementioned findings, she was instructed to stop taking Artri King and started on replacement physiologic hydrocortisone with a plan to follow up with endocrinology in the outpatient setting. She was lost to follow-up for some time and ran out of her hydrocortisone; however, once seen in clinic, she displayed no features of glucocorticoid withdrawal and was continued off of this medication while reassessing her adrenal function with repeat ACTH stimulation testing.

## 3. Discussion

In this case, it was determined that the patient's Cushingoid features were most likely caused by sustained usage of the Artri King supplement. There was no other identifiable source of exogenous glucocorticoids, including topical, inhaled, or intranasal preparations. No further biochemical testing for hypercortisolism was pursued on the basis of her compelling physical features and symptoms of Cushing's syndrome with a known source of exogenous glucocorticoids.

Her care was complicated by secondary adrenal insufficiency as demonstrated by ACTH stimulation testing. This was thought to be due to suppression of her hypothalamic-pituitary-adrenal axis by prolonged corticosteroid use. Secondary adrenal insufficiency is a common complication of steroid use, with incidence varying widely depending on steroid potency, duration, and route of administration [4]. Adrenal insufficiency can present with a wide spectrum of disease severity, including hypotension and shock in cases of adrenal crisis. It is therefore important that patients at risk for glucocorticoid withdrawal undergo a careful taper when stopping steroids. In this case, given that her previous steroid dose was unknown, she was started on physiologic hydrocortisone with a plan to attempt tapering from there rather than from a known prior dose.

Most cases of iatrogenic Cushing's syndrome are due to use of oral glucocorticoids for the treatment of chronic inflammatory diseases, however numerous cases have been reported describing iatrogenic Cushing's syndrome caused by other means. One such mechanism is by the administration of glucocorticoids by routes less likely to cause systemic glucocorticoid excess, which has been rarely reported with the use of topical, inhaled, or intranasal steroids [5–7]. Very rarely, Cushing's syndrome has been reported as a consequence of surreptitious glucocorticoid ingestion in what is known as “factitious Cushing's syndrome,” a variant of factitious disorder imposed on self [8]. Lastly, and relevant to this case, Cushing's syndrome can be caused by unwitting ingestion of substances not known to the user to contain glucocorticoids. Numerous reports exist of Cushing's syndrome caused by over-the-counter or herbal/alternative medicines used to treat various conditions [3, 9].

Review of the literature yields two previously published reports of iatrogenic Cushing's syndrome caused by unintentional ingestion of glucocorticoids contained within a Mexican supplement marketed for joint pain relief known as “Artri King.” Little information is available about this supplement online; however in April of this year the FDA published a public notification warning consumers that it contained an undeclared and unspecified amount of dexamethasone [10]. The first of these reports describes a suspected relationship between use of the Artri King supplement and a number of iatrogenic Cushing's syndrome cases in Veracruz, Mexico [11]. The other describes two separate cases of Cushing's caused by Artri King, including one with laboratory-confirmed elevation in serum dexamethasone seen on a synthetic glucocorticoid screening test [12].

The case reported here extends the literature describing iatrogenic Cushing's syndrome as a result of Artri King use. While biochemical testing for glucocorticoid excess and laboratory confirmation of the presence of dexamethasone in the serum were not available in this case, the patient's typical Cushingoid features with a long history of exposure to Artri King (and no other steroid exposures) provide convincing support to this diagnosis. More broadly, this case reinforces the value of a thorough medication reconciliation to include over-the-counter and complementary and alternative medications. While this patient's Cushingoid features were readily evident on examination, a thorough medication history provided a satisfying diagnosis with little need for additional laboratory investigation. Furthermore, this case highlights the risks inherent in the use of herbal and supplemental therapies which are largely not subject to the same strict regulatory framework as traditional medicines.

## Data Availability

The data used to support the findings of this study are included within the article.
